# Crisis and polity formation in the European Union

**DOI:** 10.1080/13501763.2024.2313107

**Published:** 2024-02-05

**Authors:** Frank Schimmelfennig

**Affiliations:** ETH Zurich, Zurich, Switzerland

**Keywords:** Capacity, community, crisis, European Union, neofunctionalism, political development

## Abstract

Ernst Haas initially formulated neofunctionalism as a theory of incremental regional polity formation, treating crises as anomalies. Subsequent revisions of the theory incorporated crises as recurring phenomena. This paper introduces a novel conceptualisation and analysis of recent European Union crises, framing them as effects of and challenges to its regulatory polity. It distinguishes between ‘failures’ and ‘attacks’, aligning them with the capacity and community-building dimensions of polity formation. Failures, rooted in capacity deficits, prompt capacity development to sustain common policies, varying with international interdependence among member states. In contrast, attacks arise from contestations of constitutive values, necessitating community demarcation through enhanced unity among defenders and exclusion of attackers. The speed and scope of demarcation depend on the attacker's membership position. Through a comparative analysis of the euro, migration, Covid, Brexit, rule of law, and Russia crises, the study illustrates and substantiates its theoretical argument.

## Introduction: Haas, neofunctionalism and integration crises

Since 2010, the European Union (EU) has been engulfed in a series of crises threatening the flagship projects of post-Cold War European integration. The euro crisis called into question the Eurozone’s monetary union. The migration crisis undermined the Schengen regime of free movement across internal EU borders and the common asylum policy. Brexit challenged the integrity of the internal market and the irreversibility of membership. The Covid-19 pandemic renewed and reinforced the economic troubles of the euro crisis and the border closures of the migration crisis. Finally, the rule of law crisis and the Russian attacks on Ukraine have cast doubt on EU enlargement as a process of democracy promotion and peaceful expansion of European governance.

Prima facie, the work of Ernst Haas seems of little relevance for analysing integration crises. Neofunctionalism started out as a theory that explained the emergence and progress of regional integration – not its crisis. Haas’s seminal study of the *Uniting of Europe* (Haas, 1958) was published when the narrow European Coal and Steel Community expanded to the European Economic Community. By contrast, when European integration produced resistance and stagnation in the 1960s and 1970s, Haas was quick to question neofunctionalism. In response to the Gaullist backlash and the intergovernmentalist critique, Haas conceded that the assumption of steady incremental integration growth had proven wrong and that a process built on pragmatic interest was prone to reversals (1967, pp. 327–328). In the mid-1970s, Haas even declared neofunctionalism ‘obsolescent’ in a situation of international ‘turbulence’, in which member states increasingly turned to extra- and interregional rather than Community-based solutions and regional integration theory was best subsumed under a general theory of international interdependence (Haas, 1975, 1976).

Considering the remarkable revival of European integration after the mid-1980s, this declaration of obsolescence was premature (Ruggie et al., 2005, p. 280). It also underestimated the potential contribution of neofunctionalism to the study of integration crisis. For one, Haas had designed neofunctionalism as a dynamic theory of polity formation with a focus on the unanticipated and unintended feedback processes of regional institution-building and their transformative ‘spillover’ potential. This focus fits well with an analysis of integration crises as particularly severe unintended consequences of integration, which not only threaten disintegration but also present a ‘creative opportunity for realizing [the] potential to redefine aims at a higher level of consensus’ (Haas & Schmitter, 1964, p. 716).

In addition, Philippe Schmitter had made ‘crisis’ a centrepiece of his revised neofunctionalism (Schmitter, 1969, 1970). Schmitter theorised ‘crisis-provoked decisional cycles’ as a recurrent feature of the integration process, compelling ‘national or regional authorities to revise their respective strategies and, collectively, to determine whether the now joint institution(s) will expand or contract’ (Schmitter, 1970, p. 842). As it progresses through these cycles, integration becomes increasingly consolidated. This understanding of regional integration as a crisis-driven process mirrors the attention that students of political development pay to crisis as ‘a sporadic, disruptive event that suddenly challenges a state’s capacity to maintain control’ and as ‘watersheds in a state’s institutional development’ (Skowronek, 1982, p. 10).

Finally, both Haas and Schmitter moved beyond the early neofunctionalist focus on spillover in their revisions. In reaction to the Gaullist backlash, Haas (1967) contrasted ‘dramatic-political’ actors and aims in the integration process with the ‘incremental-economic’ actors and aims assumed by spillover. Schmitter (1969, pp. 165–166) complemented spillover with the ‘politicization’ and ‘externalization’ mechanisms to capture internal as well as external resistance to integration.

The recent crises of the EU have revived the neofunctionalist analysis of European integration (Niemann, 2021 for a general review; Brooks et al., 2023; Lefkofridi & Schmitter, 2015; Nicoli, 2020; Niemann & Ioannou, 2015; Niemann & Speyer, 2018). These studies examine the workings of spillover and its variants driving incremental, issue-specific institution building and focus on the role of supranational actors and interest groups in the crisis response. Some additionally contribute to the debate between integration theories, contrasting or combining neofunctionalism with (liberal) intergovernmentalism and/or postfunctionalism (Becker & Gehring, 2023; Jones et al., 2016; Schimmelfennig, 2018).

While building on the crisis-specific results of this literature, this paper makes three additional contributions. First, rather than focusing on spillover, it adopts the distinction of actors, aims, and mechanisms of integration in Haas’s and Schmitter’s revisions of neofunctionalism, which the recent crisis literature has not taken up, to conceptualise two types of crises: failures and attacks. Second, it further develops the neofunctionalist perspective on regional polity formation, on which the policy-oriented neofunctionalist crisis studies have less to say. Rather than contributing to the debate between integration theories, it employs state-theoretical concepts to analyse the EU’s crisis period. Finally, it offers a comparative analysis of six recent EU crises.

Whereas EU studies has long laid to rest the analogy and teleology of modern state formation, conceptual references to varied ‘forms of state’ (Caporaso, 1996) or polity types (Schmitter, 1996) have remained as fruitful as comparisons with historical state and empire building processes (Bartolini, 2005; Marks, 2012). In this vein, recent work has assessed the EU’s crises in the tradition of Rokkanian and Tillyan approaches to state formation (Ferrera et al., 2023; Kelemen & McNamara, 2022).

Rather than subscribing to any specific state-building theory, this paper singles out two generic dimensions of polity formation: capacity building (the centralisation of authority and the creation of common resources) and community building (the creation of a common identity and the demarcation of insiders and outsiders). Moreover, in contrast to Ferrera et al. (2023) who emphasise the *compound* nature of the EU polity as the main source of crisis and framework of crisis management, this paper focuses on the EU as a *regulatory* polity. In the post-Cold War period, the regulatory EU polity massively expanded its policy portfolio beyond market integration and its membership beyond Western Europe. This leap in integration put EU capacity and community under stress. For one, newly integrated policies such as monetary policy and the free movement of persons were still designed as regulatory policies with weak supranational capacity to weather policy shocks. In addition, the deepening of integration politicised European integration domestically and mobilised nationalist voters and parties. Finally, the EU regulatory polity expanded to numerous countries that had only recently transitioned from communism and which Russia continued to regard as part of its geopolitical sphere of influence.

In the polity formation perspective of this paper, the EU crises have originated in these capacity deficits and challenges to community. It further argues that the two dimensions of political development exhibit different types of integration crises and crisis management processes. ‘Failures’ result from shocks that reveal a lack of capacity to maintain common policies. They typically generate a common interest in policy maintenance, but also distributional conflict about the costs of preservation. Depending on the intensity of policy interdependence, failures result in capacity development. By contrast, attacks originate in resistance against core values of the community. They trigger a crisis process driven by ideological disagreement about the preservation of the existing order. Attacks primarily sharpen the demarcation of the community. They increase the unity of the polity’s defenders while leading to the exclusion of the attackers. Variation in the depth and speed of community demarcation further depends on whether the attack is internal or external to the EU.

Against this background, the paper offers a comparative analysis of six recent crises of European integration. The euro, migration, and Covid-19 crises originated in failures, to which the EU responded with capacity development – most extensively in the euro and Covid crises, in which the costs of disintegration were highest and national crisis management capability was most limited. By contrast, the Brexit, Russia, and rule of law crises originated in attacks and resulted primarily in community demarcation. The speed and depth of demarcation has been highest in the external Russia crisis and lowest in the internal rule of law crisis.

## The EU regulatory polity and its expansion

The study of polity formation has focused on the modern nation-state, which became the universal model of political organisation in the twentieth century. The compound noun points to two major but distinct processes in the development of the nation-state: state building and nation building (Linz, 1993). State building, in the Weberian tradition, refers to the centralisation of territorial political authority, including the monopoly of the legitimate use of physical force, the power to extract resources and provide public goods, and the establishment of a bureaucracy. By contrast, nation building refers to the construction of a collective identity of the people living in the state’s territory, based on a shared culture and sense of belonging, and to the development of a ‘demos’. To avoid equating EU polity formation with nation-state formation, I replace state and nation building with the more open terms of capacity and community building. Capacity building in the EU refers to the allocation of legislative, executive, and judicial authority to supranational institutions and the creation of administrative, fiscal, and coercive capacity at the EU level. Community building focuses on the inclusion and exclusion of nation-states in the EU’s supranational community based on shared values and norms.

Giandomenico Majone aptly categorised the EU, as it had developed in the early 1990s, as a supranational ‘regulatory state’ (Majone, 1994, 1996). The main purpose of the regulatory polity is market integration. Its authority is designed mainly to overcome regulatory failures caused by opportunistic behaviour of the member states and the weak credibility of intergovernmental agreements. Correspondingly, its activities focus on legislative rule making and judicial rule enforcement.

The regulatory EU polity is characterised by thin capacity and thin community. In contrast to its significant rule-making and judicial powers, the EU has minor administrative, fiscal, and coercive capacity. The member states provide the administrative capacity to implement EU rules, the macroeconomic and welfare state policies that flank the EU’s market integration, and the police and military forces that ensure internal and external security (Kelemen, 2014). After the failure of the EDC in the early 1950s, NATO has provided the collective defense of most EU member states. In addition, the EU community rests mainly on a shared commitment to liberal values and norms such as human rights, democracy, the rule of law, and the market economy. Identification with ‘Europe’ lacks the traits of a thick, ethnic identity. It has remained secondary to identification with the nation(-state) for a very large majority of citizens. Values, on the one hand, and language and religion, on the other, are on opposite ends of the list of issues that most create a sense of community among EU citizens, and peace, democracy, and human rights top the list of values that best represent the EU for them.^1^

In the political development perspective of this paper, the massive ‘deepening’ and ‘widening’ of European integration in the 1990s and 2000s has in many ways pushed the limits of the regulatory polity model, without concomitantly strengthening its capacity or community, and thereby prepared the ground for the subsequent crisis period. First, the ‘1992’ internal market programme increased the intrusiveness of the regulatory polity. Negative market integration focused on removing national barriers and limited the scope of national public policies (Scharpf, 1999). By including the free movement of persons, it also reduced the member states’ ability to control the boundaries of the national community. Second, Eastern enlargement expanded the membership of the EU from 15 to 28 and beyond its traditional Western European core. In line with the regulatory polity model, EU enlargement was based predominantly on the regulatory alignment of nonmember states with EU rules (Schimmelfennig & Sedelmeier, 2004).

Moreover, in what Philipp Genschel and Markus Jachtenfuchs term the ‘regulatory seizure of core state powers’ (2014, p. 6), the EU moved beyond its original purpose of market integration. For one, it started to impose regulatory constraints on the national exercise of core state powers such as taxation, access to the welfare state, or military procurement. In addition, the EU gained own competences in the domain of core state powers – as in monetary policy, asylum policy, or internal security policies. However, the EU integrated these core state powers in a predominantly regulatory mode, without creating significant supranational administrative, fiscal, or coercive capacity (Genschel & Jachtenfuchs, 2016). Monetary union focused on the strong regulatory powers of the independent European Central Bank (ECB), weaker regulatory seizure of national budgets (in the Excessive Deficit Procedure and the Stability and Growth Pact), and the regulatory objective of price stability. The Schengen free-travel area established a common external border but left border control almost exclusively to the member states. Likewise, the common asylum policy was limited to regulating member state responsibilities for handling asylum seekers and specifying minimum standards for national asylum procedures.

The expansion of the EU regulatory polity relied implicitly on a benign, ‘fair weather’ economic, geopolitical, and domestic environment. It chimed well with the post-Cold War globalisation and democratisation trends and the dominant neoliberal policy paradigm. At the same time, the functional and territorial expansion of the regulatory polity required intergovernmental consensus, which favoured ‘least common denominator’ agreements, worked against demanding, let alone redistributive, joint capacity building, and thus rendered EU policies ‘incomplete’ by contemporary nation-state standards (Genschel & Jachtenfuchs, 2016; Jones et al., 2016; Kelemen & McNamara, 2022).

As a result, the EU arguably came to suffer from ‘regulatory polity overstretch’ in terms of citizen support, capacity development, and both territorial and policy scope. For one, the expansion of the regulatory polity eroded citizens’ ‘permissive consensus’ on integration. The more visible, intrusive, and heterogeneous EU also became more politicised domestically. European integration turned into a divisive issue in the cultural dimension of political conflict and a domain of party competition. Eurosceptic parties successfully mobilised national identities in national and European elections (Hooghe & Marks, 2009; Kriesi, 2016).

In addition, the EU lacked the supranational capacity to stabilise its new flagship regulatory regimes against policy shocks. Monetary union came without a common fiscal policy or fiscal capacity. The ECB was prohibited from providing credit facilities to member states or Community institutions. Moreover, whereas the EU had removed barriers to the integration of financial markets, it lacked the capacity to deal with ailing banks that had expanded in these markets and become too big to rescue for their home countries.

The Schengen/Dublin free-movement regime was established without a supranational capacity to protect the borders of the EU, handle asylum requests, or distribute refugees across the EU. Instead, it assigned responsibility for the policing of external borders and the processing of asylum requests to the outer member states whose national capacities are quickly overwhelmed when large numbers of asylum-seekers arrive. Moreover, open internal borders and highly diverse asylum and economic conditions incentivize ‘asylum shopping’ and illegal secondary movement of refugees.

Finally, through its enlargement and association policies, the EU expanded into a region, which had only recently transitioned to liberal democracy and market economy, and which had belonged to the Soviet/Russian state or sphere of influence during the Cold War. Yet, the EU did not build the capacity to defend its community against internal and external state detractors. Although the ‘Article 7’ procedure allows the EU to suspend rights of member states in breach of the EU’s fundamental values, it requires a highly unlikely unanimity (minus one) to determine whether such a breach has occurred. Lastly, the EU ‘civilian power’ does not have the defense capacity to protect countries in its regulatory ambit against military aggression.

In sum, in its expansion to new policies and members in the 1990s and 2000s, the EU remained faithful to its regulatory polity model. Yet this expansion tended to put further pressure on the already thin capacity and community of the regulatory polity. The EU thereby became not only more susceptible to international and domestic shocks, but also lacked the capacity and community underpinnings to prevent such shocks from developing into integration crises. The recent EU crises share an element of backlash against the expansion of the regulatory polity. In addition, however, they differ in terms of the polity formation dimension in which they originated and played out.

## Failures, attacks, and political development

According to Boin et al. (2016, p. 5), a crisis occurs if ‘a social system – a community, an organization, a policy sector, a country, or an entire region – experiences an urgent threat to its basic structures or fundamental values, which harbors many ‘unknowns’ and appears to require a far-reaching response’ (Boin et al., 2016, p. 5). In the EU context, crises can be further specified as manifest threats of disintegration – a decline in functional scope (the breakdown of common policies), territorial scope (members and associates), and/or EU authority (constitutional principles and powers) (Schmitter, 1970, p. 845; Webber, 2014, p. 342).

I further propose to distinguish two types of crises that relate to the capacity-building and the community-building dimensions of polity formation, respectively. This distinction is based on the origins of the crisis: its immediate cause or trigger. Failures originate in shocks to policies that expose capacity deficits. An urgent threat of disintegration develops when the polity lacks the instruments and resources to deal efficiently with the shock. By contrast, attacks originate in political resistance against the polity’s identity, fundamental values, and constitutional principles. Attacks thereby reveal community deficits. Whereas failures typically happen without any actor calling for disintegration or intentionally provoking the crisis, attacks consist in deliberate actions by opponents of the polity. And whereas failures are typically policy-specific, attacks are directed against core (constitutional) features of the polity.^2^ Failures are thus closest to Haas’s original spillover mechanism starting from unanticipated dysfunctions and externalities of integration, whereas attacks correspond with the politicisation and externalisation mechanisms additionally introduced by Schmitter (1969).

Because of the thin capacity and community of its regulatory polity, the EU is prone to both failures and attacks, especially as it has massively expanded its policy and geographical scope. It lacks the administrative, fiscal, and coercive competences and resources for weathering unanticipated dysfunctions or shocks, and without deeply rooted European identities and loyalties, it remains vulnerable to attacks.

**Figure 1.**
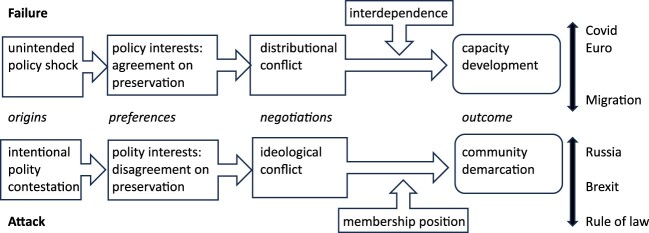
Failures, attacks, and political development.

The distinction of failures and attacks is theoretically relevant. They not only differ by the immediate crisis origins, but also exhibit distinct responses, processes, and outcomes (see Figure 1). Simply put, because failures expose unanticipated problem-solving and capacity deficits, the crisis management process focuses on capacity development. By contrast, attacks generate a process of community demarcation. I build my argument on the assumption that the main crisis actors in the EU are states or governments. EU-level failures typically only develop when vulnerable member states are overwhelmed by policy shocks, and attacks only constitute manifest threats of disintegration when they are delivered by governments. Moreover, the major decisions on capacity development and community demarcation in the EU are intergovernmental.

Member states are likely to agree on policy preservation – and the reforms required to preserve the policy – when faced with failure. First, failures result from unprovoked policy shocks to policies that the member states had supported before the crisis. Second, in line with neofunctionalist assumptions about path dependence (Pierson, 1996), the costs of policy reversal typically exceed the costs of policy preservation due to endogenous interdependence and sunk costs. In response to failures, negotiations therefore tend to focus on the distribution of preservation costs among the member states rather than on the desirability of preservation itself.

In addition, actors decide to adjust the policy and build additional capacity depending on the extent of crisis interdependence, i.e., the costs of disintegration and the efficiency of national policy. If disintegration costs are high, and national crisis management capability is low, interdependence is intense and member states are likely to accept significant costs of capacity building to preserve the policy. By contrast, if disintegration costs are low or national measures are efficient in dealing with the crisis, member states are less willing to engage in major and costly capacity building reforms. I therefore hypothesise that (1) *in failures,* (a) *crisis management focuses on capacity development,* and (b) *capacity development increases with interdependence.*

By contrast, attacks are rooted in fundamental disagreement about polity preservation. The attackers aim at disintegration, i.e., they seek to deviate from fundamental values of the polity, abolish constitutional principles, and even withdraw from or break up the EU. Moreover, whereas the response to failures is driven mostly by issue-specific cost–benefit calculations, ideological polity interests prevail in attacks. Actors who prioritise ideological polity interests exhibit reduced sensitivity to policy costs and losses. Ideology-laden crisis interactions create a polarising dynamic, which increases cohesion within the opposed camps but drives attackers and defenders ever further apart.

The communal and ideological nature of attacks reduces the chances of persuasion or compromise. It is unlikely that the attackers (re)adopt the identity and values of the supranational community. It is equally unlikely that the defenders agree to redefine their supranational community and polity – be it based on internalised community values and identities, be it out of fear that constitutional compromise could unravel the polity and destroy the benefits they reap from it. Both attackers and defenders also regularly forgo policy benefits of compromise for the sake of preserving the integrity and autonomy of their respective national and supranational communities. Finally, the regulatory polity does not possess and is unlikely to build the coercive capacity necessary to force attackers to comply – in the way that states coerce separatists, rebels, and external aggressors. Attacks therefore typically result in community demarcation, which consists in a dual move towards more unity and inclusion among the community’s defenders, on the one hand, and the exclusion of the attackers, on the other.

The speed and scope of community demarcation depend not only on the depth of the identity and ideological rift, but also on the initial membership position of the attacking governments. Attacks from within the membership are more difficult to handle for the defenders than attacks from the outside. Internal attackers wield stronger institutional power and tend to cause higher costs of exclusion for the defenders. The defenders are therefore not only more reluctant to act swiftly and forcefully against the attacker; they also find it more difficult to generate the majorities or consensus to exclude a member state from the EU’s institutional and policy benefits. In the case of external attacks, or if an internal attacker is determined to leave the EU, a unified front of the (remaining) member states is easier to form, and it is easier to agree on joint action, due to typically lower dependence on outsiders. Accordingly, (2) *in attacks* (a) *crisis management focuses on community demarcation* and (b) *community demarcation is sharper and faster in external attacks.*

Figure 1 gives an overview of the argument and categorises six recent crises of the EU. The subsequent sections provide a comparative analysis grouped by crisis type. The analysis starts by justifying why the six crises qualify as ‘manifest threats of disintegration’ and why they are best classified as either failures or attacks. The analysis covers all cases that satisfy the crisis definition and feature prominently in the EU crisis literature.^3^ At the same time, the six crises are sufficiently independent from each other to compare them as analytically separate cases. Importantly, none of the crises originated in response to another, and the crisis management process was mainly tailored to the specific challenges of each failure and attack.

To illustrate the theoretical argument empirically, the comparative analysis focuses on the commonalities and differences of failures and attacks in terms of origins, preferences, negotiations, and outcomes, and it describes how variations in capacity building (among the failure cases) and in community demarcation (between the attacks) correspond with variations in interdependence and membership position, respectively. It does not aim to explain under which conditions latent tensions, conflicts, or challenges become manifest threats of disintegration. Finally, the analysis does not build on original data, nor can it provide detailed case studies of the individual crises.^4^ Its main aim is to make conceptual and theoretical sense of the EU’s crisis period.

## Failure and capacity building: the euro, migration, and Covid-19 crises

Three EU crises are best classified as failures: the euro crisis (2009–2015), the migration (or refugee or Schengen) crisis (2015/16), and the Covid-19 pandemic (2020–2022). In each case, the crisis was triggered by an external shock to an integrated policy that first overwhelmed the most vulnerable member states and then developed into a manifest threat of disintegration. The euro crisis was the final link in a chain reaction initiated by the US subprime mortgage crisis in 2007, which triggered a global financial crisis, followed by a European banking crisis. The state rescue of systemically relevant banks caused a sovereign debt crisis in several member states, putting their continued participation in the Eurozone at risk. In addition, the euro crisis was rife with speculation about the survival of the common currency. German chancellor Angela Merkel even warned, ‘if the Euro fails, […] Europe fails’.^5^

In the migration crisis, armed conflict and political repression in the Middle East led to a surge in migrant numbers and overburdened the Mediterranean ‘frontline’ countries. Subsequently, seven Schengen members reintroduced controls at their internal EU borders, and the Dublin rules for the allocation and conditions of asylum procedures were suspended (by Germany) or ignored (by many others), threatening the EU’s border and asylum regime with breakdown (Scipioni, 2018). Finally, the Covid-19 pandemic reached Europe when the EU member states had not yet fully recovered from the euro and migration crises. It caused a recession that even exceeded that of the euro crisis and massive unilateral restrictions to cross-border movements. Again, the Covid-19 pandemic quickly generated ‘doubts about the survival of the European Union itself’ (van Middelaar, 2021, p. 2).

None of these crises resulted from a targeted external attack against the EU’s supranational policies, but each revealed a lack of EU crisis management capacity. Financial market actors did not intend to bring down the common currency in the euro crisis. Rather, the EU did not have the competence or the resources to financially rescue banks or governments. In the migration crisis, refugees or their countries of origin did not aim at unravelling the EU or its asylum policy. However, the EU had highly limited means for supporting overburdened frontline member states financially or administratively. Nor did the EU have a mandate to alleviate their burden by reallocating asylum seekers. The Covid-19 pandemic combined a health crisis with an economic crisis. Whereas health policy had become subject to EU regulatory seizure (Greer, 2006), legal competence and health resources remained almost exclusively with the member states who quickly approached limits in providing protective equipment and intensive care. In the economic sphere, it soon became clear that the capacities the EU had built during the euro crisis were insufficient to counter the even higher recession and debt levels. The common origin in unintended, and largely unanticipated or underestimated, policy shocks, and the common exposure of capacity deficits of the EU regulatory polity, qualify these crises as failures.

In all three cases, crisis management was geared towards creating the joint capacity required to prevent disintegration and overcome the crisis. Member governments generally agreed to maintain (their participation in) the euro, the internal market, the Schengen free-travel area – and, ultimately, the EU. They disagreed, however, on the measures needed to achieve this goal. Generally, this disagreement had its roots in asymmetrical interdependence, i.e., the uneven affectedness and national crisis management capability of the member states – and in the concomitant uneven willingness to engage in risk and burden sharing and the costly development of joint capacity. Consequently, the EU-level crisis management process exhibited distributional conflict, in which the member states pursued issue-specific cost-minimizing interests.

In the euro crisis, fiscally healthy surplus countries propagated domestic austerity, fiscal discipline, stricter budget surveillance and credit- as well as conditionality-based financial support to minimise their own financial contribution. By contrast. the deficit countries called for fiscal expansion and transfers, e.g., in the form of Eurobonds or unlimited purchases of debt by the ECB (Armingeon & Cranmer, 2018; Schimmelfennig, 2015). Despite a higher symmetry of affectedness between the member states (Ferrara & Kriesi, 2022), the Covid-19 pandemic saw a return of the divide on the issues of joint bonds, conditional vs. unconditional, and loan- vs. grant-based financial support (Becker & Gehring, 2023).

Finally, in the migration crisis, the member states sought to minimise the burden of processing asylum requests and hosting refugees. Government preferences mainly reflected how much the member states were affected by the arrival or destination of migration flows, which mirrored geography and national asylum conditions. Whereas the heavily affected frontline and destination states called for the redistribution of refugees, transit and by-stander countries opposed relocation (Biermann et al., 2019). Note that while immigration is often linked to the community-building dimension of polity formation, this has not been the dominant issue in the EU migration crisis.

Based on these conflicting distributional preferences, member states entered negotiations on policy reform and capacity development. By contrast, community demarcation did not play a significant role. Member states did not seek exit from the integrated policy regimes; calls for the exclusion of individual countries (e.g., regarding Greece’s membership in the Eurozone and Schengen) remained sporadic and inconsequential. In the end, the Eurozone and Schengen membership remained intact, and the pandemic crisis instruments applied to all member states.

All three failures have resulted in significant additional EU capacity. In the euro and pandemic crises, interdependence was sufficiently high to produce major capacity development. In the migration crisis, however, disintegration costs were comparatively low and national crisis management capability was comparatively high. Consequently, supranational capacity development has remained at a lower level.

In the euro crisis, the no-bailout policy was replaced with institutions providing deficit countries with common public credit. In addition to the European Stability Mechanism (ESM), a permanent international financing institution with a capital stock of €700 billion, the ECB adopted new bond-purchasing and low-interest credit facilities that have transformed it into a de facto ‘lender of last resort’, committing itself to ‘whatever it takes to preserve the euro’ (ECB President Mario Draghi in 2012) and generating a ‘quasi-fiscal capacity’ (Schelkle, 2014). Finally, the EU created a supranational ‘banking union’, which assigned the supervision of systemically significant banks to a new supranational authority at the ECB and built up a Single Resolution Fund (SRF) to resolve failing banks.

In the pandemic, the member states authorised the Commission to temporarily raise €100 billion and provide loans to member states in support of active labour market measures (SURE). In addition, the SRF was insured by a ‘common backstop’. The major innovation, however, was the Next Generation EU (NGEU) €750 billion recovery fund. In quantitative terms, NGEU effectively increased the EU budget by more than two thirds without an increase in direct member state contributions. In qualitative terms, it marked the first time the EU engaged in collective ‘borrowing for spending’ (i.e., budget transfers) rather than the ‘borrowing for lending’ of the euro crisis.

In the health domain, the EU expanded its initially limited competence and capacity with the aim of building a European Health Union. In addition to strengthening the mandates of existing agencies, it established HERA, the Commission’s Health Emergency Preparedness and Response Authority in 2021, which centralises the administrative capacity of the EU and commands a cumulative budget of €30 billion (Brooks et al., 2023).

In response to the migration crisis, the administrative and coercive capacity of the EU was enhanced. In 2016, Frontex was renamed the European Border and Coast Guard Agency with expanded tasks in the protection of the EU’s external borders; its budget has grown from €143 million in 2015 to €845 million in 2023; and its staff has increased from 309 in 2015 to more than 2100 in 2023. Moreover, in 2021, the EU upgraded its European Asylum Support Office to the European Union Agency for Asylum (EUAA) to support the member states administratively in applying EU asylum law. Finally, the operational role and powers of the EU’s border agencies were strengthened. Under certain circumstances, they may even intervene directly on the territory of the member states to help enforce EU border governance (Fernández-Rojo, 2021).

Yet, capacity development in the migration crisis is significantly different from the other failures. First, in the euro and Covid-19 crises, the EU built its fiscal capacity at the height of the crises, and to prevent them from escalating further, whereas administrative and coercive capacities in response to the migration crisis increased gradually and after the crisis had already subsided. Second, the EU’s financial commitments in the euro and Covid crises exceeded those in the migration crisis by several orders of magnitude. Third, the member states consented to major constraints on decision-making autonomy during the euro crisis. They accepted national debt brakes, the ex-ante control of national budgets, and quasi-automatic sanctions against excessive deficits. No such constraints were agreed on in the migration crisis. The Schengen Border Code, which accords the member states significant leeway in re-introducing border controls, was not tightened, and a reform of the Common European Asylum System – with more equitable burden sharing as its centrepiece – was blocked. Finally, the gains in authority of the EU’s border agencies have remained significantly more limited than those that the ECB acquired or of which it availed itself.

In line with Hypothesis 1b, the variation in the extent of capacity development can be explained by the size of disintegration costs and the national capability deficits exposed by the crises (Schimmelfennig, 2018). Whereas estimates put the immediate exit costs from the Eurozone at a prohibitive 20–50 percent share of member state GDP^6^, the annual ‘costs of non-Schengen’ would not have amounted to more than 0.2 percent of GDP (Bertelsmann-Stiftung, 2016; Lilico et al., 2016). In addition, national crisis mitigation capability differed vastly. In the euro crisis, without massive external financial support, the most affected countries would not have been able to avert sovereign default while remaining in the Eurozone. In the migration crisis, however, even vulnerable member states could effectively use national means to reduce affectedness: re-establish border controls, build fences, stop registering asylum-seekers, and transport migrants to their neighbours’ borders. Moreover, in the migration crisis, the EU was able to externalise problem-solving in a way that it could not in the euro and Covid crises, e.g., through agreements with and financial support for Turkey and several Northern African countries. This ‘outsourcing’ of crisis management alleviated the EU partly from investing in its own capacity (Genschel & Jachtenfuchs, 2018; Slominski & Trauner, 2018).

## Attacks and community building: the Brexit, rule of law, and Russia crises

The Brexit, rule of law, and Russia crises originated in the resistance of nationalist actors against core principles of European integration and the loss of national control resulting from supranational integration. They are therefore best classified as attacks. In 2013, UK Prime Minister David Cameron promised to renegotiate the terms of UK membership and hold an exit referendum to deflect the increasing electoral threat of the United Kingdom Independence Party (UKIP) and win the support of Eurosceptics in his own Conservative Party. Both UKIP and the Eurosceptic Tories were motivated by principled opposition to the loss of national sovereignty through European integration. Concerns about immigration based on the internal market freedom of movement amplified their opposition (Clarke et al., 2017; Evans & Menon, 2017). When the Cameron government announced the in-or-out referendum for June 23, 2016, domestic resistance turned into a manifest threat of disintegration.

Russia first attacked Ukraine militarily in 2014, after the ‘Euromaidan’ protests had ousted the Russia-friendly Yanukovych government and the new Ukrainian government had signed an association agreement with the EU. In 2022, Russia escalated the conflict to an all-out war to regain full control over Ukrainian territory. Even though the war does not constitute a direct military attack on the EU, it goes against the integrity of an EU-associated country, the expansion of the EU regulatory space, and the liberal-democratic values of the EU. Russian autocratic imperialism clashes with the EU’s liberal supranational order. Moreover, it raised fears that the restoration of Russia’s sphere of influence would not stop with Ukraine. In February 2022 at the latest, the threat of disintegration had become manifest.

Finally, the rule of law crisis resulted from the strategy of authoritarian governments, most dramatically in Hungary (since 2010) and Poland (after 2015), to gradually constrain internal checks of their rule, including the independence of the judiciary. Because the rule of law is a fundamental value of the EU, and a prerequisite of a functioning supranational regulatory polity, governments that systematically weaken the rule of law at home do not only undermine national democracy but also constitute a crisis for the EU. Whereas the Russia and rule of law crises are ongoing, the Brexit crisis ended with the exit of the UK and the conclusion of the Trade and Cooperation Agreement (TCA) in 2020.

None of the attacks resulted from failures of integrated policies. The UK had been a willing complier with EU internal market regulations and had even opened its labour market to immigrants from the new Eastern member states immediately after accession in 2004. The EU’s Neighborhood and Eastern Partnership policies had gradually paved the way for the association of Ukraine and other Eastern European countries. And the new member states had introduced major reforms strengthening judicial independence and the rule of law ahead of joining the EU. The three crises were not triggered by unintended shocks revealing capacity deficits. On the contrary, they were led by governments who perceived EU policies to be exceedingly intrusive and clash with national sovereignty.

Both attackers and defenders have pursued ideological polity interests in the three crises – and they generally have done so at the expense of issue-specific material benefits. Fundamental disagreement on polity principles has stood in the way of compromise or persuasion. Rather, the crisis management process has been characterised by a dual dynamic, in which growing unity among the defenders of the EU’s values, and progressive alienation and exclusion of the attackers, have led to an increasingly sharp demarcation of the boundaries of the EU community.

In the Brexit negotiations, both UK and EU negotiators insisted on the integrity of their respective polities, thereby leaving little room for compromises that would have minimised policy costs. Where the UK insisted on national sovereignty, the EU defended the indivisibility of the internal market and its four freedoms (Schimmelfennig, 2022). If the EU had granted the UK a formal opt-out from the freedom of movement in the ‘New Settlement’ negotiations, the outcome of the referendum might well have been different given the centrality of the issue and the defection of leading Conservatives to the Leave camp when the EU had only consented to minor concessions (Evans & Menon, 2017, p. 50). On the other hand, the UK government ruled out a customs union with the EU or participation in the internal market along the lines of the European Economic Area, both of which would have been compatible with the Brexit vote and economically preferable to a free-trade agreement. Yet, any movement by Prime Minister Theresa May in this direction (as in the Chequers Plan of July 2018) was thwarted by the ‘hard Brexiters’ in her party. Within a few years, the UK went from internal market membership to the TCA, and thus the most minimal EU economic integration of any West European non-member state.

At the same time, the EU managed to preserve and strengthen internal unity. The EU-27 were quick to unite behind a common frame and strategy for the negotiations and maintained a common front throughout (Hillion, 2018; Laffan & Telle, 2023). In addition, most Eurosceptic parties, which had welcomed the Brexit vote and hoped for a mounting exit wave, saw their expectations disappointed and stopped advocating exit from the Eurozone or the EU (Chopin & Lequesne, 2021).

In contrast to the UK, the democratic backsliders do not aim at exit from the EU. Therefore, the rule of law crisis has developed a gradual *internal* demarcation dynamic. Despite unlikely success, the European Commission triggered the Article 7 procedure against Poland in 2017; in 2018, the European Parliament (EP) did the same against Hungary. In 2019, the European People’s Party (EPP) suspended the membership of Fidesz; in March 2021, Fidesz left the EPP, pre-empting expulsion. In addition, the defenders of the community have moved from social opprobrium to financial sanctions. In 2020, the EU passed a regulation establishing rule of law conditionality for the EU budget, which was first triggered against Hungary. Moreover, the Commission has withheld approval of Hungary’s and Poland’s plans for payments from NGEU funds, totalling more than €30 billion. In addition, the Court fined Poland for not complying with its rule of law rulings. When the Polish government refused to pay the fine, the Commission decided to withhold budget payments. Yet, the EU’s increasing assertiveness, the sharpening of its sanctioning instruments, and the growing financial pressure have not induced the backsliders to comply beyond merely cosmetic changes – even though both countries are the largest net beneficiaries of the EU budget, Poland in absolute terms and Hungary in relation to the population. Only domestic reversals, as in the Polish elections of 2023, hold the promise of ending the attack.

In the Russia crisis, both the EU and the Russian government have incurred major economic costs in the pursuit of their polity interests. Whereas the EU was Russia’s most important trading partner overall, the EU depended on Russia for more than 40 percent of its gas and coal imports as well as a quarter of its oil imports. After the 2014 annexation of Crimea, EU sanctions had remained limited. In response to the 2022 invasion, however, the EU has imposed unprecedented sanctions including travel bans, asset freezes, import restrictions, and embargoes on Russia. In return, Russia cut off most EU countries from gas supplies. In line with the expectation of fast and sharp community demarcation towards external attackers, the EU has largely suspended its institutional and transactional ties with Russia. Ukraine has moved in the opposite direction. In 2022, the EU offered the country candidate status for membership. It opened its borders for refugees, lowered barriers for trade, and massively increased its military and fiscal support (Freudlsperger & Schimmelfennig, 2023). After two decades, in which the EU had kept its Eastern neighbourhood in a grey zone of association without a membership perspective, the Russian invasion has triggered a clear-cut divide between community insiders and outsiders.

In sum, all three crises support the expectation that attacks produce demarcation and exclusion rather than compromise or persuasion, despite considerable economic incentives to the contrary. Because the EU regulatory polity does not possess and cannot mobilise the coercive capacity to stop the attacks and force the attackers to comply, it relies on its the regulatory powers to exclude its detractors from its market and policies. The EU’s efforts to reaffirm its values, and to mobilise its institutions and instruments in their defense, have increased the unity and support among the community defenders but failed to preserve or strengthen community with the UK, Hungary, Poland (under the PiS government), and Russia.

In addition, and in line with Hypothesis 2b, the comparison shows that external attacks tend to produce faster and deeper community demarcation than internal attacks. In the rule of law crisis, a fully internal attack, it took the defenders a long time to unite against the attack and take determined action. The ‘slow-burning’ (Boin et al., 2016) nature of the crisis is part of the explanation. The backsliding governments have undermined national democracy gradually and did not demand any formal change to the EU’s norms and rules initially. Moreover, however, the determination of the backsliders to remain in the EU, and the lack of a formal exclusion procedure, make it hard to achieve a clear demarcation. By contrast, the external Russian attack triggered swift action leading to the almost complete breakdown of ties once it had escalated into a full-blown invasion of Ukraine. Brexit is an in-between case. While the Cameron government intended to keep the UK in the EU, both sides negotiated and agreed on a ‘New Settlement’ for UK membership; after the Brexit vote, however, the relationship quickly disintegrated.

In contrast to the failures, capacity development has been neither a strong focus nor a major outcome of EU crisis management. In responding to the attacks, the EU has predominantly operated within its narrow regulatory polity paradigm. Attacks have not led to the creation of new organisations and allocation of major additional administrative, fiscal, and coercive resources to the EU. Attacks mainly challenge the EU to reaffirm its values, create and preserve unity, and find determination to use its extensive regulatory powers against the attackers.

In the Brexit crisis, EU capacity development was limited to providing a team and a process for the effective conduct of negotiations (Laffan & Telle, 2023). The EU defended the integrity of its internal market both by reaffirming the indivisibility of the four freedoms and by clarifying the boundary between members and nonmembers, but it did not change the rules of the internal market or build supranational capacity. Additional supranational powers and resources were not needed for effective demarcation.

The rule of law crisis has not produced major capacity change either. The EU did not introduce a mechanism for excluding member states or reform its Art. 7 procedure for suspending their rights. Treaty changes requiring unanimity are generally unsuitable for coping with internal attacks. The problem was rather that EU institutions have long been reluctant to use their considerable powers against backsliding member states (Kelemen, 2023). When they started to use these powers, the instruments – judicial enforcement, rule of law conditionality, and budget cuts – remained regulatory in nature. No major additional fiscal or administrative resources were transferred to the EU for the purpose.

In the Russia crisis, the EU has also used predominantly its existing regulatory powers, rules, and decision-making mechanisms, e.g., for sanctions against Russia and Belarus, the international protection of refugees, and the facilitation of imports from Ukraine. In addition, the member states redirected and topped up existing resources, mainly the European Peace Facility (EPF) established in 2021 and funded by extra-budgetary member state contributions, to support Ukraine militarily. In October 2022, the EU agreed on a novel EU Military Assistance Mission (EUMAM), but the actual training of Ukrainian armed forces takes place in the member states. Finally, the EU did not move to abolish the Council’s unanimity rule in matters of foreign and security policy.

## Conclusions

Neofunctionalism conceives of regional integration as a process of polity formation. Ernst Haas’s early version of the theory assumed an incremental spillover process propelled by the functional benefits of task expansion to overcome unanticipated dysfunctions of integration. The revisions of neofunctionalism by Ernst Haas and Philippe Schmitter not only brought in ‘crisis’ as a recurring feature of the polity formation process, but also went beyond the spillover mechanism (producing policy learning and task expansion among ‘incremental-economic’ actors) to introduce politicisation and externalisation by ‘dramatic-political’ actors as additional mechanisms.

This paper builds on the neofunctionalist legacy to theorise and analyse the recent crises of the EU. It not only adopts a polity formation perspective but also builds on the distinction of integration mechanisms in the revised versions of the theory. Specifically, it distinguishes failures and attacks as crisis responses to capacity and community deficits of the post-Cold War expansion of the EU regulatory polity. It thereby goes beyond the numerous analyses of EU crises inspired by neofunctionalism, including the prominent ‘failing forward’ framework (Jones et al., 2016). For one, they continue to focus on the spillover mechanism (and its variants) and therefore have been more often and more successfully applied to the failure crises than to the Brexit and rule-of-law attacks (Conant, 2021; Jones et al., 2021; Schimmelfennig, 2022). In addition, they have contributed to the debate between integration theories rather than the study of polity formation.

In the polity formation perspective, the findings of this paper provide support to the neofunctionalist expectation of self-stabilizing integration dynamics. While significant supranational integration is likely to generate failures and attacks, it also creates powerful stakeholders and path dependencies that prevent disintegration and work in favour of capacity development and community demarcation. Crisis-driven political development has turned the EU into a better resourced, more robust, and ‘maturing’ regulatory supranational polity (Caporaso et al., 2015; Freudlsperger & Schimmelfennig, 2022; Rhinard, 2023).

It has not, however, brought about a structural break in the EU’s polity model. In the capacity-building dimension, the crisis-induced additional steps of integration were mostly incremental and patchy fixes, which equipped the EU regulatory polity with just sufficient capacity to prevent it from being overwhelmed and undermined (Jones et al., 2016). Moreover, capacity development has followed an issue-specific rather than a transversal logic. Additional capacity has been tailored to the specific crisis at hand with specialised agencies, targeted funds, and often temporary instruments. The crises have not changed the constitutional structure, the institutional architecture, or the overall horizontal or vertical division of competences. In the community-building dimension of polity formation, the EU has proven resilient, without clear signs of supranational identity or loyalty formation. Overall, popular support for the EU has typically suffered from failures, recovered after the end of the failures, and increased under the impact of attacks. But it has not exceeded pre-crisis levels.^7^ In neofunctionalist terminology, the EU appears to be amid the ‘transformative’ stage of crisis-induced political development (Schmitter, 1970, p. 865) – but not on the verge of ‘transcendence’.

## Notes

1 See Fligstein (2008), Special Eurobarometer 508/2021 (Values and Identities of European Citizens), and the most recent Standard Eurobarometers (European Citizenship) 98 and 99 (Winter 2022/2023 and Spring 2023), available at https://europa.eu/eurobarometer/surveys/browse/all/series/4961.

2 Let me reemphasize that the distinction of the two types is based on the *immediate cause* triggering the crisis. Going further back in history, the lack of joint (fiscal or coercive) capacity to effectively deal with policy shocks may be rooted in a community deficit of the EU, and resistance against EU community norms may have resulted from ‘failure’ to prevent such resistance. Moreover, attacks may subsequently lead to policy failure, and the management of failures may create resistance against the EU polity (as it did during the euro crisis in southern member states subject to austerity).

3 See the recent compilations in Rhinard et al. (2023) and Riddervold et al. (2021), which add a few other ‘crises’ that do not fit the definition of manifest threats of disintegration, such as the ‘climate crisis’, the ‘Trump crisis’, and a general ‘legitimacy crisis’ of the EU.

4 For general reference, see the relevant chapters in the edited volumes of Rhinard et al. (2023) and Riddervold et al. (2021).

5 https://www.reuters.com/article/euro-merkel-20100513-idDEBEE64C04I20100513, 13 May 2010.

6 https://drive.google.com/file/d/0B2bjqNteRwUoMzhmYjYzYTAtOTY0Yy00ZDg0LWI4ZjctYzQ1OTUwOGNlYmFm/view?resourcekey=0-MonU4fFevK0j5dbbekFy_w. Cf. Bertelsmann-Stiftung, 2012.

7 On the development of trust in the EU, see the latest Standard Eurobarometer results at https://europa.eu/eurobarometer/surveys/detail/3052.

## References

[CIT0001] Armingeon, K., & Cranmer, S. (2018). Position-taking in the Euro crisis. *Journal of European Public Policy*, *25*(4), 546–566. 10.1080/13501763.2016.1268642

[CIT0002] Bartolini, S. (2005). *Restructuring Europe. Centre formation, system building and political structuring between the nation-state and the European Union*. Oxford University Press.

[CIT0003] Becker, M., & Gehring, T. (2023). Explaining EU integration dynamics in the wake of COVID-19: A domain of application approach. *Journal of European Public Policy*, *30*(2), 334–353. 10.1080/13501763.2022.2027000

[CIT0004] Bertelsmann-Stiftung. (2012). Economic impact of Southern European member states exiting the eurozone. *Future Social Market Economy Policy Brief*. https://www.bertelsmann-stiftung.de/fileadmin/files/BSt/Presse/imported/downloads/xcms_bst_dms_36656__2.pdf.

[CIT0005] Bertelsmann-Stiftung. (2016). *Departure from the Schengen Agreement. Macroeconomic impacts on Germany and the countries of the European Union* (Global Economic Dynamics, Issue. https://www.bertelsmann-stiftung.de/fileadmin/files/BSt/Publikationen/GrauePublikationen/NW_Departure_from_Schengen.pdf.

[CIT0006] Biermann, F., Guerin, N., Jagdhuber, S., Rittberger, B., & Weiss, M. (2019). Political (non-)reform in the euro crisis and the refugee crisis: A liberal intergovernmentalist explanation. *Journal of European Public Policy*, *26*(2), 246–266. 10.1080/13501763.2017.1408670

[CIT0007] Boin, A., t’Hart, P., Stern, E., & Sundelius, B. (2016). *The politics of crisis management. Public leadership under pressure* (2nd ed.). Cambridge University Press.

[CIT0008] Brooks, E., de Ruijter, A., Greer, S. L., & Rozenblum, S. (2023). EU health policy in the aftermath of COVID-19: neofunctionalism and crisis-driven integration. *Journal of European Public Policy*, *30*(4), 721–739. 10.1080/13501763.2022.2141301

[CIT0009] Caporaso, J. A. (1996). The European Union and forms of state: Westphalian, regulatory or post-modern? *Journal of Common Market Studies*, *34*(1), 29–52. 10.1111/j.1468-5965.1996.tb00559.x

[CIT0010] Caporaso, J. A., Kim, M., Durrett, W. N., & Wesley, R. B. (2015). Still a regulatory state? The European Union and the financial crisis. *Journal of European Public Policy*, *22*(7), 889–907. 10.1080/13501763.2014.988638

[CIT0011] Chopin, T., & Lequesne, C. (2021). Disintegration reversed: Brexit and the cohesiveness of the EU27. *Journal of Contemporary European Studies*, *29*(3), 419–431. 10.1080/14782804.2020.1714560

[CIT0012] Clarke, H., Goodwin, M., & Whiteley, P. (2017). *Brexit: Why Britain voted to leave the European Union*. Cambridge University Press.

[CIT0013] Conant, L. (2021). Failing backward? EU citizenship, the Court of Justice, and Brexit. *Journal of European Public Policy*, *28*(10), 1592–1610. 10.1080/13501763.2021.1954061

[CIT0014] Evans, G., & Menon, A. (2017). *Brexit and British politics*. Polity Press.

[CIT0015] Fernández-Rojo, D. (2021). *Eu migration agencies. The operation and cooperation of Frontex, EASO and EUROPOL*. Edward Elgar.

[CIT0016] Ferrara, F. M., & Kriesi, H. (2022). Crisis pressures and European integration. *Journal of European Public Policy*, *29*(9), 1351–1373. 10.1080/13501763.2021.1966079 36032420 PMC9397131

[CIT0017] Ferrera, M., Kriesi, H., & Schelkle, W. (2023). Maintaining the EU's compound polity during the long crisis decade. *Journal of European Public Policy*. 10.1080/13501763.2023.2165698 PMC10896164 38414981

[CIT0018] Fligstein, N. (2008). *Euroclash: The EU, European identity, and the future of Europe*. Oxford University Press.

[CIT0019] Freudlsperger, C., & Schimmelfennig, F. (2022). Transboundary crises and political development: why war is not necessary for European state-building. *Journal of European Public Policy*, *29*(12), 1871–1884. 10.1080/13501763.2022.2141822

[CIT0020] Freudlsperger, C., & Schimmelfennig, F. (2023). Rebordering Europe in the Ukraine War: community building without capacity building. *West European Politics*, *46*(5), 843–871. 10.1080/01402382.2022.2145542

[CIT0021] Genschel, P., & Jachtenfuchs, M. (2014). Introduction: Beyond market regulation. Analysing the European Integration of Core State Powers. In P. Genschel, & M. Jachtenfuchs (Eds.), *Beyond the regulatory polity? The European integration of core state powers* (pp. 1–23). Oxford University Press.

[CIT0022] Genschel, P., & Jachtenfuchs, M. (2016). More integration, less federation: The European integration of core state powers. *Journal of European Public Policy*, *23*(1), 42–59. 10.1080/13501763.2015.1055782

[CIT0023] Genschel, P., & Jachtenfuchs, M. (2018). From market integration to Core State Powers: The Eurozone crisis, the refugee crisis and integration theory. *Journal of Common Market Studies*, *56*(1), 178–196. 10.1111/jcms.12654

[CIT0024] Greer, S. L. (2006). Uninvited Europeanization: Neofunctionalism and the EU in health policy. *Journal of European Public Policy*, *13*(1), 134–152. 10.1080/13501760500380783

[CIT0025] Haas, E. B. (1958). *The uniting of Europe: Political, social and economic forces, 1950–1957*. Stanford University Press.

[CIT0026] Haas, E. B. (1967). The uniting of Europe and the uniting of Latin America. *Journal of Common Market Studies*, *5*(4), 315–343. 10.1111/j.1468-5965.1967.tb01153.x

[CIT0027] Haas, E. B. (1975). *The obsolescence of regional integration theory*. Institute of International Studies.

[CIT0028] Haas, E. B. (1976). Turbulent fields and the theory of regional integration. *International Organization*, *30*(2), 173–212. 10.1017/S0020818300018245

[CIT0029] Haas, E. B., & Schmitter, P. C. (1964). Economics and differential patterns of political integration: Projections about unity in Latin America. *International Organization*, *18*(4), 705–737. 10.1017/S0020818300025297

[CIT0030] Hillion, C. (2018). Withdrawal under Article 50 TEU: An integration-friendly process. *Common Market Law Review*, *55*(Special Issue), 29–56. 10.54648/COLA2018059

[CIT0031] Hooghe, L., & Marks, G. (2009). A postfunctionalist theory of European Integration: From permissive consensus to constraining dissensus. *British Journal of Political Science*, *39*, 1–23. 10.1017/S0007123408000409

[CIT0032] Jones, E., Kelemen, R. D., & Meunier, S. (2016). Failing forward? The Euro crisis and the incomplete nature of European Integration. *Comparative Political Studies*, *49*(7), 1010–1034. 10.1177/0010414015617966

[CIT0033] Jones, E., Kelemen, R. D., & Meunier, S. (2021). Failing Forward? Crises and patterns of European integration. *Journal of European Public Policy*, *28*(10), 1519–1536. 10.1080/13501763.2021.1954068

[CIT0034] Kelemen, R. D. (2014). Building the new European state? Federalism, core state powers, and European integration. In P. Genschel, & M. Jachtenfuchs (Eds.), *Beyond the regulatory polity? The European integration of core state powers* (pp. 211–229). Oxford University Press.

[CIT0035] Kelemen, R. D. (2023). The European Union’s failure to address the autocracy crisis: MacGyver, Rube Goldberg, and Europe’s unused tools. *Journal of European Integration*, *45*(2), 223–238. 10.1080/07036337.2022.2152447

[CIT0036] Kelemen, R. D., & McNamara, K. R. (2022). State-building and the European Union: Markets, War, and Europe's Uneven Political Development. *Comparative Political Studies*, *55*(6), 963–991. 10.1177/00104140211047393

[CIT0037] Kriesi, H. (2016). The politicization of European integration. *Journal of Common Market Studies*, *54*, 32–47. 10.1111/jcms.12406

[CIT0038] Laffan, B., & Telle, S. (2023). *The EU Response to Brexit. United and effective*. Palgrave.

[CIT0039] Lefkofridi, Z., & Schmitter, P. C. (2015). Transcending or descending? European integration in Times of Crisis. *European Political Science Review*, *7*(1), 3–22. 10.1017/S1755773914000046

[CIT0040] Lilico, A., Leghari, S. L., & Hegg, M. (2016). *The Cost of Non-Schengen: the Impact of Border Controls within Schengen on the Single Market*. https://www.europarl.europa.eu/RegData/etudes/STUD/2016/581383/EPRS_STU%282016%29581383_EN.pdf.

[CIT0041] Linz, J. J. (1993). State building and nation building. *European Review*, *1*(4), 355–369. 10.1017/S1062798700000776

[CIT0042] Majone, G. (1994). The rise of the regulatory state in Europe. *West European Politics*, *17*(3), 77–101. 10.1080/01402389408425031

[CIT0043] Majone, G. (1996). *Regulating Europe*. Routledge.

[CIT0044] Marks, G. (2012). Europe and its empires: From Rome to the European Union. *Journal of Common Market Studies*, *50*(1), 1–20. 10.1111/j.1468-5965.2011.02218.x

[CIT0045] Nicoli, F. (2020). Neofunctionalism revisited: Integration theory and varieties of outcomes in the Eurocrisis. *Journal of European Integration*, *42*(7), 897–916. 10.1080/07036337.2019.1670658

[CIT0046] Niemann, A. (2021). Neofunctionalism. In M. in Riddervold, J. Trondal, & A. Newsome (Eds.), *The Palgrave handbook of EU Crises* (pp. 115–133). Palgrave Macmillan.

[CIT0047] Niemann, A., & Ioannou, D. (2015). European integration in times of crisis: A case of neofunctionalism. *Journal of European Public Policy*, *22*(2), 196–218. 10.1080/13501763.2014.994021

[CIT0048] Niemann, A., & Speyer, J. (2018). A neofunctionalist perspective on the “European Refugee Crisis”: The case of the European Border and Coast Guard. *Journal of Common Market Studies*, *56*(1), 23–43. 10.1111/jcms.12653

[CIT0049] Pierson, P. (1996). The path to European Integration. A historical institutionalist analysis. *Comparative Political Studies*, *29*(2), 123–163. 10.1177/0010414096029002001

[CIT0050] Rhinard, M. (2023). The EU’s crisis logics: Towards a maturing polity? In M. Rhinard, N. Nugent, & W. Paterson (Eds.), *Crises and challenges for the European union* (pp. 354–368). Bloomsbury.

[CIT0051] Rhinard, M., Nugent, N., & Paterson, W. (Eds.). (2023). *Crises and challenges for the European union*. Bloomsbury.

[CIT0052] Riddervold, M., Trondal, J., & Newsome, A. (Eds.). (2021). *The Palgrave handbook of EU crises*. Palgrave Macmillan.

[CIT0053] Ruggie, J. G., Katzenstein, P. J., Keohane, R. O., & Schmitter, P. C. (2005). Transformations in world politics: The intellectual contributions of Ernst B. Haas. *Annual Review of Political Science*, *8*, 271–296. 10.1146/annurev.polisci.8.082103.104843

[CIT0054] Scharpf, F. W. (1999). *Governing in Europe: Effective and democratic?* Oxford University Press.

[CIT0055] Schelkle, W. (2014). Fiscal integration by default. In P. Genschel, & M. Jachtenfuchs (Eds.), *Beyond the regulatory polity. The European integration of core state powers* (pp. 105–123). Oxford University Press.

[CIT0056] Schimmelfennig, F. (2015). Liberal intergovernmentalism and the euro area crisis. *Journal of European Public Policy*, *22*(2), 177–195. 10.1080/13501763.2014.994020

[CIT0057] Schimmelfennig, F. (2018). European integration (theory) in times of crisis. A comparison of the euro and Schengen crises. *Journal of European Public Policy*, *25*(7), 969–989. 10.1080/13501763.2017.1421252

[CIT0058] Schimmelfennig, F. (2022). The Brexit puzzle: polity attack and external rebordering. *West European Politics*. 10.1080/01402382.2022.2132448 PMC11018062 38628813

[CIT0059] Schimmelfennig, F. (2023). Polity attacks and policy failures: The EU polycrisis and integration theory. In M. Roos, & D. Schade (Eds.), *The EU Under Strain* (pp. 27–50). De Gruyter.

[CIT0060] Schimmelfennig, F., & Sedelmeier, U. (2004). Governance by conditionality: EU rule transfer to the candidate countries of Central and Eastern Europe. *Journal of European Public Policy*, *11*(4), 661–679. 10.1080/1350176042000248089

[CIT0061] Schmitter, P. C. (1969). Three neo-functional hypotheses about international integration. *International Organization*, *23*(1), 161–166. 10.1017/S0020818300025601

[CIT0062] Schmitter, P. C. (1970). A revised theory of international integration. *International Organization*, *24*(4), 836–868. 10.1017/S0020818300017549

[CIT0063] Schmitter, P. C. (1996). Imagining the future of the Euro-Polity with the help of new concepts. In G. Marks, F. W. Scharpf, P. C. Schmitter, & W. Streeck (Eds.), *Governance in the European Union* (pp. 121–150). Sage.

[CIT0064] Scipioni, M. (2018). Failing forward in EU migration policy? EU integration after the 2015 asylum and migration crisis. *Journal of European Public Policy*, *25*(9), 1357–1375. 10.1080/13501763.2017.1325920

[CIT0065] Skowronek, S. (1982). *Building a new American state: The expansion of national administrative capacities, 1877–1920*. Cambridge University Press.

[CIT0066] Slominski, P., & Trauner, F. (2018). How do member states return unwanted migrants? The strategic (non-)use of “Europe” during the migration crisis. *Journal of Common Market Studies*, *56*(1), 101–118. 10.1111/jcms.12621

[CIT0067] van Middelaar, L. (2021). *Pandemonium: Saving Europe*. agenda.

[CIT0068] Webber, D. (2014). How likely is it that the European Union will *dis*integrate? A critical analysis of competing theoretical perspectives. *European Journal of International Relations*, *20*(2), 341–365. 10.1177/1354066112461286

